# Validation of the Ottawa Ankle Rules in Iran: A prospective survey

**DOI:** 10.1186/1471-227X-6-3

**Published:** 2006-02-16

**Authors:** Shahram Yazdani, Hesam Jahandideh, Hossein Ghofrani

**Affiliations:** 1 Education Development Center, Shaheed Beheshti University of Medical Sciences, Evin, Tabnak St., Tehran, Iran

## Abstract

**Background:**

Acute ankle injuries are one of the most common reasons for presenting to emergency departments, but only a small percentage of patients – approximately 15% – have clinically significant fractures. However, these patients are almost always referred for radiography. The Ottawa Ankle Rules (OARs) have been designed to reduce the number of unnecessary radiographs ordered for these patients. The objective of this study was to validate the OARs in the Iranian population.

**Methods:**

This prospective survey was done among 200 patients with acute ankle injury from January 2004 to April 2004 in the Akhtar Orthopedics Hospital Emergency Department. Main outcome measures of this survey were: sensitivity, specificity, positive predictive value, negative predictive value, and likelihood ratios (positive and negative) of the OARs.

**Results:**

Sensitivity of the OARs for detecting 37 ankle fractures (23 in the malleolar zone and 14 in the midfoot zone) was 100% for each of the two zones, and 100% for both zones. Specificity of the OARs for detecting fractures was 40.50% for both zones, 40.50% for the malleolar zone, and 56.00% for the midfoot zone. Implementation of the OARs had the potential for reducing radiographs by 33%.

**Conclusion:**

OARs are very accurate and highly sensitive tools for detecting ankle fractures. Implementation of these rules would lead to significant reduction in the number of radiographs, costs, radiation exposure and waiting times in emergency departments.

## Background

**Figure 1:**
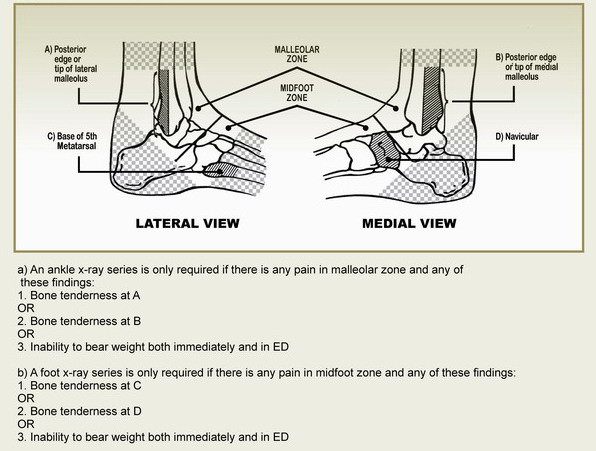
Ottawa Ankle Rules^(4)^.

Ankle injuries are one of the most common reasons for presenting to orthopedics emergency department. However, although only a few of these patients – approximately 15% – have a significant clinical fracture, radiography is performed on almost all patients, without having any positive diagnostic result in 85% of cases [1-6]. Steill et al started a multi-stage project in 1992 for the first time in order to find a way to face this challenge and to provide decision-making criteria in using radiography for ankle injuries. By developing the Ottawa Ankle Rules (OARs), Steill et al attempted to help physicians rapidly recognize patients who have no fractures, improve the application of decision rules to "common clinical practice", and reduce the radiographs ordered by physicians by a rate of 26.4% without causing an adverse effect on health care quality [6-10]. The rules were based only on evaluating bone tenderness and weight bearing (Fig. 1).

Scientific reports about clinical decision rules are increasingly published in medical literature. These rules (*a*) are decisional tools resulting from research projects rather than consensus-based clinical practice guidelines; (*b*) are coordinated results of three or more variables in clinical history, physical examination and simple tests; and (*c*) are used in determining the diagnosis, prognosis and possible responses in each individual patient. These tools help the physician to effectively challenge his/her uncertainty in clinical decision-making. In addition, using these rules can enhance the physician's efficiency, which is a must in the current situation where health care systems are increasingly calling for more cost-effective methods in clinical practice [11].

There have been several attempts to validate the OARs in different countries [12-21]. In their systematic review, Bachman et al [22] showed that the sensitivity of The OARs range from 96.4% (95% confidence interval: 93.8–98.6%) in some studies to 99.6% (95% confidence interval: 98.2–100%) in others. Also, specificity ranges from 47.9% (interquartile range: 42.3–77.1%) to 26.3% (19.4–34.3%).

Despite these successful results, however, there are other studies that could not validate the OARs [23-25]. In addition, since no specific standard is used for the diagnosis and treatment of ankle injuries in Iran – especially in public teaching hospitals – it appears that there is a tendency toward "defensive medicine" among physicians. Therefore, in view of the high prevalence of ankle injuries as well as difficulties experienced in the current trend of radiography requisitions, and considering the unique features of the OARs, validation of these rules in an Iranian population has been evaluated.

## Methods

This prospective study was performed in a 3-month period from January-April 2004 on 237 patients presenting to Akhtar Orthopedics Hospital Emergency Department with ankle pain or tenderness following a blunt trauma. The definitions of ankle zones based on Steill studies are as follows:

1. Malleolar zone: 6 cm of the distal fibula and tibia as well as the talus bone

2. Midfoot zone: Navicular, cuboid, cuneiforms, anterior process of calcaneus and the base of the fifth metatarsal bone.

Patients who were less than 16 years of age or pregnant, those with injuries of more than seven days, those referring for re-evaluation, and those with multiple trauma or decreased level of consciousness were excluded from study.

Patients were physically examined and evaluated regarding the 8 clinical variables included in the OARs. Each patient's data was recorded and coded. All patients were referred for standard radiography of the malleolar zone, midfoot zone or both according to the presence of pain or tenderness in one or both of these zones. Radiography results were interpreted by an orthopedics surgery resident who had not visited or examined the patients.

For statistical analysis, SPSS for Windows V. 10.0 was used. Sensitivity, specificity, positive and negative likelihood ratio, and positive and negative predictive value with a 95% confidence interval were calculated.

## Results

After excluding 36 patients according to the exclusion criteria and 1 patient for inability to cooperate, 200 patients were evaluated. The patients' mean age was 31.86 ± 15.95 years. Most patients were young adults, 60% having less than 30 years of age. Among the examinees, 52.5% (105 cases) were male and 47.5% were female. Fifty one percent of patients reached the hospital within 7 hours of injury (Table 1). Types of treatment provided for the patients are listed in Table 2. Of all patients, 142 had injuries only in malleolar zone, 37 were injured only in midfoot zone and 21 had injuries in both zones (Table 3).

**Table 1:** Patients' Characteristics.

	**No**	**Percent**
**Sex**		
Male	105	52.50%
Female	95	47.50%

**Age mean(SD), year**	31.86(± 15.95)	

**Mechanism of injury**		
Sports activities	49	24.50%
Descending stairs	31	15.50%
Falling in pot-holes	25	12.50%
twisting ankle during casual walking	20	10.00%
Direct trauma	17	8.50%
Falling down	16	8.00%
Tripping over obstacles	13	6.50%
Others	29	14.50%

**Time of Arrival to Emergency**		
<3 h	56	28.00%
4–7 h	46	23.00%
8–12 h	23	11.50%
13–24 h	47	23.50%
25–48 h	19	9.50%
>48 h	9	4.50%

**Fractures**	37	18.50%
*Malleolar zone*	23	62.16%
Lateral malleol	15	40.54%
Medial malleol	5	13.51%
Bimalleolar	3	8.11%
Calcaneus	0	0%
Talus	0	0%
*Midfoot zone*	14	37.84%
Base of 5^th ^metatarsal	13	35.14%
Navicular	0	0%
Cuboid	1	2.70%
Coneiforms	0	0%

**Treatments**		
Short leg splint	111	55.50%
Short leg cast	42	21.00%
Conservative management	38	19.00%
Surgery	5	2.50%
Others	4	2.00%

N = 200

**Table 2:** Types of treatment performed on ankle injury patients

Type of injury	Type of treatment				
	
	Short leg splint	Short leg cast	Conservative management	Surgery	Others
Fracture (N = 37)	9 (24.32%)	19 (51.35%)	1 (2.70%)	5 (13.51%)	3 (8.11%)
Ligamentous (N = 163)	102 (62.58%)	23 (14.11%)	37 (22.70%)	0 (0%)	1 (0.61%)

**Table 3:** Rate of injuries and conformity of the OARs results with diagnostic feature

	Malleolar zone		Midfoot zone		Concomitant injury in both zones		Total	
	
	OAR+	OAR-	OAR+	OAR-	OAR+	OAR-	*OAR+*	OAR-
Fracture	21	0	12	0	4	0	*37*	0
No Fracture	72	49	11	14	14	3	*97*	66
Total	93	49	23	14	18	3	134	66

N= 200

Causing mechanisms for injuries included sport activities (49 patients, 24.5%), descending stairs (31 patients, 15.5%), falling in pot-holes (25 patients, 12.5%), twisting ankle during casual walking (20 patients, 10%), direct trauma (17 patients, 8.5%), falling down (16 patients, 8%), tripping over obstacles (13 patients, 5.6%) and others (29 patients, 14.5%).

Of this number, 37 cases (18.5%) had fractures, of which 23 cases (62.16%) were in the malleolar zone and 14 cases (37.84%) in the midfoot zone. Therapeutic interventions included short leg splint (111 patients, 55.50%), short leg cast (42 patients, 21.00%), conservative management (38 patients, 19.00%), surgical operation (5 patients, 2.5%) and others (4 patients, 2.00%).

As shown in Table 3, the OARs sensitivity in detecting fractures was 100% (95% CI: 85.30–100%) for those with isolated malleolar injuries (142 patients, 21 fractures), 100% (95% CI: 73.33–100%) for isolated midfoot injuries (37 patients, 12 fractures), and 100% (95% CI: 32–100%) for concomitant fracture of both zones (21 patients, 4 fractures). The sensitivity of the OARs was also calculated to be 100% (95% CI: 91.82–100%) in overall evaluation (200 patients, 37 fractures).

The overall specificity, specificity for the malleolar zone, the midfoot zone and injuries to both zones were 40.50% (95% CI: 32.87–48.11%), 40.50% (95% CI: 31.62–49.37%), 56.00% (95% CI: 35.09–76.91%), and 17.65%, respectively. Negative predictive values for malleolar, midfoot and overall rate of fractures were 100% (95% CI: 93.86–100%), 100% (95% CI: 77.43–100%), and 100% (95% CI: 95.46–100%), respectively. Negative likelihood ratio was nil for all three evaluations.

The positive predictive value of the OARs was 22.58% (95% CI: 13.92–22.58%) for malleolar zone fractures, 52.17% (95% CI: 30.09–74.26%) for midfoot fractures and 27.61% (95% CI: 19.94–35.28%) in overall evaluation. The positive likelihood ratio was 1.68, 2.27 and 1.68 for the malleolar zone, the midfoot zone and the overall evaluation, respectively.

Negative predictive value, negative likelihood ratio, positive predictive value and positive likelihood ratio for concomitant injuries were calculated to be 100%, 0, 22.22% and 1.21, respectively.

## Discussion

Several studies have been performed since 1981 to develop clinical decision-making rules for using radiographs in ankle injuries [2,4,5,10,12-22].

The OARs were designed, reviewed and validated by its Canadian inventors, and used in various clinical settings. Their simplicity in application and memorization [26] has made them a very powerful tool to decrease radiology department referrals and to save cost and time. In addition, these rules have been successfully and favorably validated in the US, the UK, France, the Netherlands, Greece, Spain, Australia and Hong Kong.

Without validation, however, even well defined decision-making rules are not suitable for application in all clinical settings, for three reasons. First, predictive rules resulting from a study on a patient population may only demonstrate an accidental relation between presumed predictive factors and outcomes. Thus, there may be a quite different set of predictive factors in other groups of patients.

Second, the relations between predictive factors and the population under study, physicians using the rules or other aspects of study design, may have unique and specific features. This may also invalidate clinical decision-making rules in new circumstances.

Third, physicians may not be able to use decision-making rules comprehensively or perfectly because of some feasibility problems in a specific clinical setting. Therefore, all decision-making rules need to be validated.

Moreover, some study results [23-25] have rejected the generalizablity of the OARs, although these studies had considerable methodological errors or did not use *real *rules [28,29]. Therefore, considering the differences in human populations and also in physicians' behavior, validation of the OARs was considered in this study.

Traditionally, immobilization, functional treatment – i.e., an early mobilization protocol with the use of external support – and surgical treatment are three main treatments for acute lateral ankle ligament ruptures. However, in several reviews, functional treatment and early mobilization – especially with lace-up supports- have been preferred to immobilization in a cast or surgical operation [30]. Since our results showed that cast and splint are still used for the treatment of ligamentous injuries, it seems necessary to take effective action in modifying this improper trend.

It is estimated that more than 5 million radiographs are ordered annually in Northern America, costing about 500 million US dollars. It must be noted that multiple low-cost tests such as plain radiographs can be as much a financial burden to health system as high-tech, high-cost but fewer medical interventions [31]. In addition, patients are more satisfied if they do not have to go under radiography [10].

According to the present study, of about 70 patients presenting each day to the Akhtar Orthopedics Hospital Emergency Department, approximately 20% have ankle injuries. Thus, of a roughly estimated 25,500 presentations each year, 5100 are only for ankle injuries. Based on the tariffs confirmed by the Iranian Ministry of Health for public hospital services, anterior-posterior and lateral radiographs of the ankle zone cost about 24000 Rls (2.80 US dollars), while the cost for a radiograph of the foot zone is 26600 Rls (3.10 US dollars). Moreover, in most ankle injuries, both radiographs are ordered. If only 33% of radiographs could be avoided by using the OARs, savings would reach up to 85,200,000 Rls (more than 10000 US dollars) each year (while the official fee for a general practitioner visit is 14400 Rls [1.70 US dollars], this would be a considerable amount). Also, we should add to this figure the indirect costs saved by reducing the time patients spend in the hospital. It is obvious how enormous the savings would be if these decision-making rules were to be used at the level of a medical university or an entire country. These savings seem to be most needed in developing countries such as Iran.

OARs application, however, has some limitations and obstacles. Would all emergency physicians agree to treat their patients without taking a radiograph? Would they take the legal responsibility in case of a possible fracture?

Some studies showed that even after attending a one-hour training program on the OARs and despite having a very good opinion towards the subject, physicians did not use the OARs [32]. In addition, the rate of radiograph reduction in current practice may not be as anticipated. This could be due to the patients' anxiety or the physicians' obsessiveness to order radiographs even when the required criteria are not met.

It should also be noted that currently, patients might not all accept the physician's avoidance from ordering a radiograph for ankle injuries, and think of it as the doctor's lack of knowledge or ignorance. Unfortunately, there is a widespread tendency among patients to use various diagnostic tools. This is also one of the challenges for using such rules.

The current study faced some limitations. The relatively low number of cases made it difficult to generalize the results to other medical centers and the entire Iranian population. In addition, because we did not have any case of calcaneus, talus, navicular or cuboid fractures, the achieved results may not be perfect in view of all fractures in this zone. Inter-observer reliability among different groups -attending physicians, residents, and interns- was also not determined.

Referring all patients for radiography and the subsequent danger of radiation exposure was not an ethical problem, because it is currently the *routine *procedure performed for *all *patients.

## Conclusion

Our study proved that the OARs have the same results in the Iranian population as in the original study and the majority of other investigations. The sensitivity of these rules was 100% for diagnosing ankle and midfoot fractures, and application of these rules significantly reduced the number of radiographs by approximately 33%. Thus, OARs application can not only decrease the number of radiology department referrals, but also can reduce costs and radiation exposure and save time for hospital staff and patients

Suggestions for further evaluation include assessing the OARs' validity with more samples; assessing the OARs' validity in different medical centers, populations and by various treatment staff with different levels of clinical skill and expertise; studying physicians' attitude about and acceptance of these rules in the clinic setting; evaluating real changes resulting from application of the OARs; and evaluating patients' and physicians' satisfaction in case of using the OARs or other such rules.

## Competing interests

The author(s) declare that they have no competing interests.

## Authors' contributions

ShY had a substantial role in the conception and design of the study.

HJ had the main role in data collection, analysis and interpretation, and preparing the draft of the manuscript.

HG had been involved in data interpretation and revised the manuscript critically.

All authors have approved the final version of the document to be published.

**Table 4:** Statistical characteristics of Ottawa Ankle Rules

	Sensitivity	Specificity	Likelihood ratio(+)	Likelihood ratio(-)	Predictive value(+)	Predictive value(-)
Both zones	100%	40.50%	1.68	0	27.61%	100%
Malleolar zone	100%	40.50%	1.68	0	22.58%	100%
Midfoot zone	100%	56.00%	2.27	0	52.17%	100%
Concomitant injuries of both zones	100%	17.65%	1.21	0	22.22%	100%

## Pre-publication history

The pre-publication history for this paper can be accessed here:


